# Experimental Analysis of the Efficiency and Usability of Tylke Versus Magill Forceps

**DOI:** 10.7759/cureus.48857

**Published:** 2023-11-15

**Authors:** Norris C Talbot, Patrick M Luther, Noah J Spillers, Steven J Alexander, Arthur J Saus, George M Jeha, Laine N Rogers, Giustino Varrassi, Shahab Ahmadzadeh, Sahar Shekoohi, Elyse M Cornett, Alan D Kaye

**Affiliations:** 1 School of Medicine, Louisiana State University Health Sciences Center, Shreveport, USA; 2 Physiology, Louisiana State University Health Sciences Center, Shreveport, USA; 3 Anesthesiology, Louisiana State University Health Sciences Center, Shreveport, USA; 4 School of Medicine, Louisiana State University Health Sciences Center, New Orleans, USA; 5 Pain Medicine, Paolo Procacci Foundation, Rome, ITA

**Keywords:** trapezius, deltoid, brachioradialis, nasotracheal intubation, nasal intubation, tylke forceps, magill forceps

## Abstract

Introduction: The procedure of nasotracheal intubation (NI) has long been performed utilizing the Magill forceps as developed by Sir Ivan Magill in the 1920s. While used for nearly a century, several serious patient safety concerns remain including torn tube cuffs, vocal cord trauma, and inefficient tube placement. The Tylke forceps have been developed as a modification to the largely unchanged form of Magill forceps.

Methods: In the present investigation we compared the efficacy, number of clasps, and muscle activation involved in NI using the Tylke forceps versus the Magill forceps in previously untrained individuals.

Results: Tylke forceps showed faster successful NI over the standard Magill forceps at an average intubation time of 6.54s vs. 13.73s, respectively. Tylke forceps also had fewer clasps per intubation over the Magill. The trapezius, deltoid, and brachioradialis muscle activation was also compared in Tylke vs Magill forceps intubation trials. Tylke forceps required less lower muscle activation in the brachioradialis and trapezius over the Magill forceps with Tylke forceps resulting in higher deltoid muscle activation.

Conclusion: Tylke forceps were more efficacious and reduced the number of clasps over the Magill forceps when used in successful NI with different muscle activation patterns.

## Introduction

Nasotracheal intubation (NI) remains an accepted method of airway management and is an essential skill for practicing anesthesiologists [1]. NI involves picking up the endotracheal tube in the oropharynx using forceps and advancing it into the trachea under vision with direct laryngoscopy. The Magill forceps, virtually unchanged since their development in 1920, remain the conventional instrument of choice for the procedure, despite several significant drawbacks that can cause airway trauma, damage to the nasotracheal tube, and delays in the procedure. In the present investigation, therefore, we considered whether the newer Tylke forceps have no difference in outcomes and ergonomics of performing endotracheal intubation when compared to the standard Magill forceps. We compared muscle activity, number of attempts, and time required to accomplish NI using the Magill forceps versus the Tylke forceps. In the present investigation, we used a non-invasive electromagnetic sensor approach (Delsys) to record the activity of various muscle groups in the right arm of the anesthetist performing the intubation. These data points suggest that Tylke forceps represent a simpler and more rapid approach for NI, which uses a different approach and group of muscles to perform the procedure.

## Materials and methods

This study was reviewed and approved by the LSUHSC-S (Louisiana State University Health Sciences Center in Shreveport) Institutional Review Board (study 00000960, “Evaluation of Tylke Forceps vs. Magill Forceps Use”). All participants provided informed consent and had the procedure explained in detail prior to beginning the study. All participants were free to leave the study at any time. All participants declared they were in good health, and no identifying health information was recorded. All participants self-identified as "right-handed" individuals.

Our study included 20 intubation procedures performed on an anesthesia practice mannequin. The 20 total intubations were split into 10 with Tylke forceps and 10 with Magill forceps. This study involved 10 medical students from the institution to perform the intubation process while we collected our data based on their trials. The study required each participant to perform an endotracheal intubation once with the Tylke and Magill forceps. In these trials, prior to procedural testing, electromagnetic sensors were affixed to the trapezius, deltoid, and brachioradialis muscles. Placed directly on the skin, the individual sensors would record the muscle activation of their respective locations. These readings are displayed as an electromyography (EMG) recorded for the entire span of each intubation procedure. In these trials, the time of each intubation was recorded on a digital timer. Each run was timed from the moment a tool was positioned and entered the oral cavity until the participant successfully completed the intubation on the mannequin. The validity of each intubation procedure was confirmed by filling the endotracheal tube with air and visualizing the mannequin’s lungs inflating. Throughout each procedure, participants were tracked for each time they had to clasp (grasp) the tube. For example, if a participant only clasped the tube on the initial grab, then the data would represent one total clasp. Otherwise, the participant announced each release and reclasping of the endotracheal tube to researchers and recorded.

Importantly, all 10 medical students declared they had no prior experience with intubation or had no experience with the Tylke or Magill forceps. Furthermore, upon each participant’s arrival and prior to the procedure, we would provide a detailed explanation of NI. After the participant understood the procedure, a demonstration was done with a laryngoscope tool and both the Tylke and Magill forceps. Once the participant felt comfortable with the procedure, we allowed him or her to practice NIs with both tools. In these practice runs, the inflation of the lungs was checked each time, and every participant was permitted to practice until he or she felt comfortable with both tools. After the participant had successfully intubated with both the Tylke and Magill forceps during the test run, the sensors were attached, and the time to intubation, number of clasps, and muscle activation were recorded. Furthermore, the muscle activation was recorded as an EMG from which lower muscle activation was taken as the root mean square (RMS) of the wave. The RMS is defined as the root mean square of recorded EMG data. We measured the RMS with the same software that recorded the EMG. Boundaries were set between the initial activation and the cessation of the signal. This method was done in order to ensure RMS was accurately attained during the procedure alone and to minimize unwanted “noise” from the calculations. This value is used to measure the average activation of the muscle. RMS can be used to assess the power of a signal, and therefore indicate the overall general activity of the muscle [2].

During the timed trial for each intubation, the participants were instructed to position the laryngoscope before the timer and sensors were recorded. This part permitted us to not gauge the effectiveness of the participants with the scope and forceps but solely with forceps. Once the scope was correctly positioned, the time was recorded upon moving towards the oral cavity with the forceps until successful intubation.

## Results

**Figure 1:**
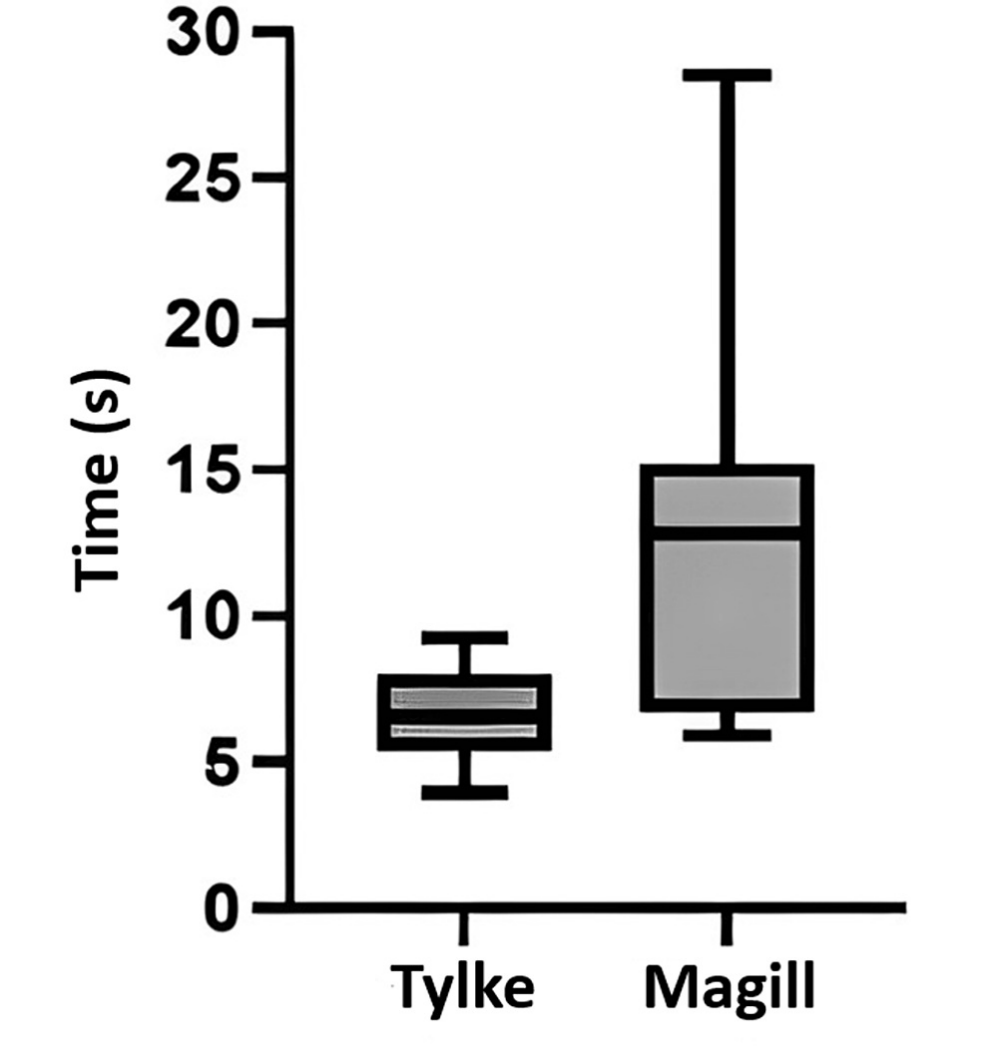
Statistical analysis of time to intubation The graph compares the time period between Tylke and Magill forceps in successfully achieving intubation. Each time was recorded from the entrance into the oral cavity until the successful placement of the intubation tube.

The finalizations of the data reported on the time to intubation, grabs of the endotracheal tube, and muscle activation of the trapezius, deltoid, and brachioradialis for each test run of the Tylke and Magill forceps. The time trial for both the Tylke and Magill was recorded in seconds from the entrance into the oral cavity until the participant successfully completed the procedure, as confirmed by lung inflation. For the Tylke forceps, the time ranged from 3.95s to 9.25s, with an average of 6.54s. The standard deviation was 1.69s, and we found a standard error of 0.53. Furthermore, the Magill forceps recorded times ranged from 5.92s to 28.51s, averaging 13.73s. The standard deviation was 8.23, and the standard error was 2.60s. Analyzing the data, the comparison between the Tylke and Magill averages were, respectively, 6.54s and 13.73s, with a significance of p<0.05 between the recorded times. This data is summarized in Figure 1.

**Figure 2:**
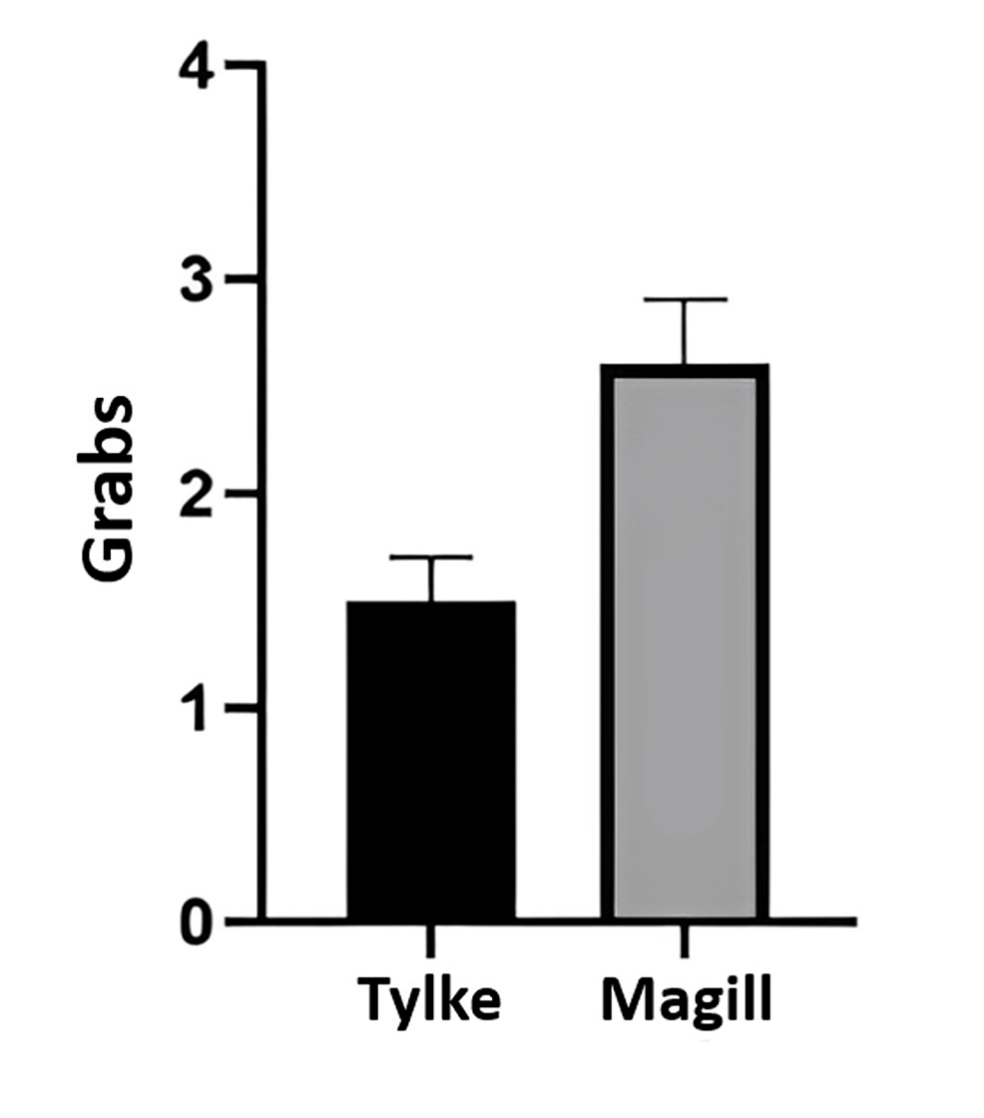
Average number of grabs for Tylke and Magill forceps The graph demonstrates the total average amount of grabs, including the standard error. Note that the minimum number of grabs possibly recorded is 1.

While the Tylke approach is designed as a quicker tool, it also showed significant results in terms of the number of clasps per procedure. These data were recorded as the total number of times the endotracheal tube was clasped, including the initial grab of the tube. For the Tylke forceps, the number of grabs ranged from 1 to 3 total grabs with an average of 1.5 ± 0.7071 (with standard deviation) grabs. Moreover, the Magill forceps provided a range of 1 to 5 total grabs with an average of 2.6 1.0750 ± 0.3399 (standard error) grabs with a significance of p<0.05, demonstrated in Figure 2.

**Figure 3:**
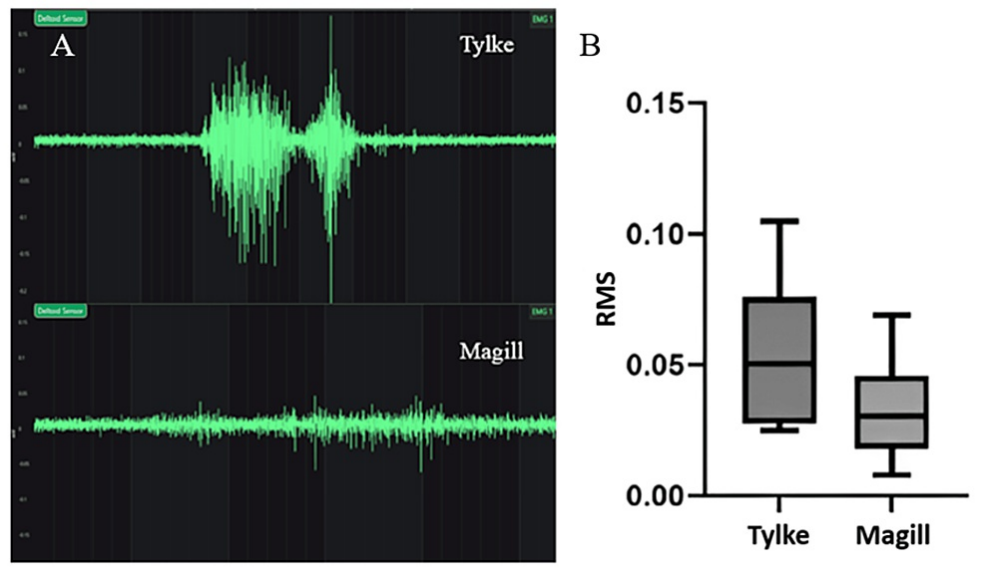
RMS data and EMG example for Tylke and Magill forceps An individual recording example of muscle activation is seen in (A). The graph in (B) represents the RMS data provided by electromagnetic sensors attached to the deltoid muscle. The RMS value represents the level of muscle activation in a participant. The value is specific to the individual’s potential for the force of muscular activation. The graph illustrates the comparison between Tylke and Magill muscle activation in the deltoid. The EMGs are separately recorded from the same participant using both tools. The y-axis is set in increments of 0.05 for both EMGs to demonstrate the difference. EMG: Electromyography; RMS: Root mean square

Furthermore, the sensors recorded muscular activation at the brachioradialis, deltoid, and upper trapezius. The upper trapezius and forearm were activated less with the Tylke tool. However, the data was not significant, comparing the activation between the forearm and upper trapezius. However, the EMGs of the deltoid relayed statistically significant results. This data was recorded based on the RMS. The RMS for the Tylke instrument had a range of 0.025 to 0.105 with an average of 0.0507. The standard deviation was 0.0269, and the standard error was 0.0085. The RMS for the Magill instrument had a range of 0.008 to 0.069 with an average of 0.044. The standard deviation was 0.0173, and the standard error was 0.0189. With regards to RMS, each participant was compared with both tools, and the ranges may be attributed to differences in the potential for muscle force applied by participants. The range of RMS is a result of the difference in total muscle strength and stature between participants rather than between tools. The participants varied in strength and stature, resulting in a wider RMS range. The comparison between the two RMS can be seen in Figure 3. Included in this figure is one participant’s recorded EMG in which the RMS was taken from muscle stimulation to the cessation of muscle stimulation.

## Discussion

NI, first described in 1902 by Kuhn, and refined and popularized in the 1920s by Rowbotham and Magill has changed very little since its introduction [1,3-5]. Commonly used for dental, oropharyngeal, and maxillofacial operations, NI allows for the delivery of anesthetic gases while providing a clear surgical field without the obfuscation of intraoral anatomy [1,6-16].

In 1920, Stanley Rowbotham described a unique method for the administration of intratracheal anesthesia via the nasal route [17-19]. This procedure involved the passage of an endotracheal tube through the nares into the nasopharynx, visualization of the tube within the oropharynx by means of a laryngoscope, and the advancement of the tube into the glottic opening using a guiding rod. Shortly thereafter, Ivan Magill developed specialized forceps to facilitate the procedure [20,21]. Magill’s forceps, constructed with a bend to clear the field of vision and a serrated inner surface on the tongs of the forceps to grasp the endotracheal tube, have remained virtually unchanged since their creation and are still the conventional instrument of choice for NI [6,20,22-24].

Although the Magill forceps have demonstrated utility for facilitating NI for many decades, they are not without their disadvantages. From an ergonomics viewpoint, the Magill forceps require the intubating anesthetist to assume a posture that is different from the normal posture assumed during oropharyngeal intubation. That is, the arm must be abducted from the body at a nearly 90-degree angle in order to correctly grasp the endotracheal tube and guide it into the glottic opening. Second, the serrated inner surface on the tongs of the Magill forceps, which grasp the endotracheal tube, can easily tear the cuff of the tube during its placement if it is not grasped far enough posteriorly. In addition, the design of the forceps demands that the endotracheal tube be regripped as it is advanced, which may prove difficult if the tube is slippery. Last, the bend in the forceps forces the user to position the wrist at an angle which induces more strain on the wrist.

In recent years, the Tylke forceps have emerged as a contemporary alternative to the conventional Magill forceps for NI. The concept for the Tylke forceps came about when the inventor, Dr. James Tylke, became frustrated with the efforts of using Magill forceps during the intubation of 14 sequential mandibular fracture cases on young healthy males over a single weekend. Following design modifications from the initial design concept, the Tylke forceps became patented in 2008.

**Figure 4:**
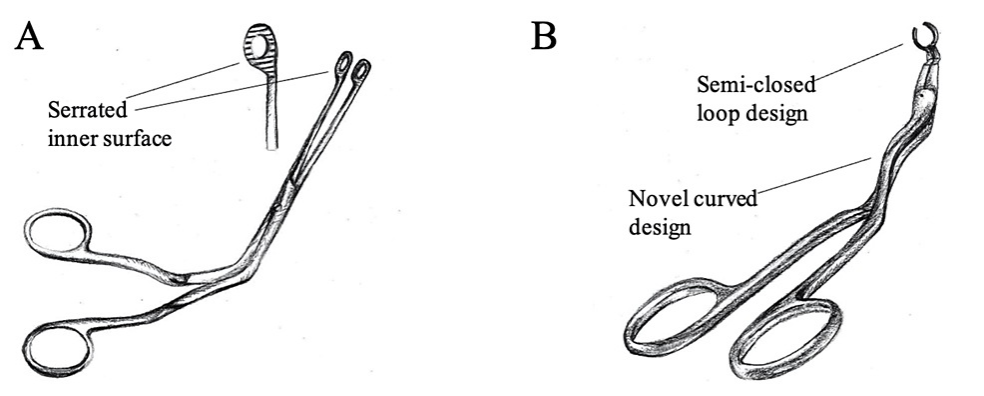
Illustration of Magill forceps (A) and Tylke forceps (B) The Magill forceps feature a serrated inner surface on the distal end of the tongs. The Tylke forceps are instead constructed with a semi-closed loop design, which reduces the risk of damaging the endotracheal tube and eliminates the need to regrip the tube as it is advanced. The Tylke forceps are also constructed with a novel set of angled bends, offering unobstructed visualization of the vocal cords. The illustration was originally created by those involved with this project.

The Tylke forceps represent an entirely different intubation approach for accomplishing NI. The curves on the Tylke handle have been designed with a different set of angled bends, offering an unobstructed visualization of the vocal cords, while allowing the arm of the intubating anesthetist to remain comfortably at the side of the body rather than being abducted away from the body during the intubation process. Instead of the serrated inner surface of the Magill forceps, which grasp the endotracheal tube and creates a risk of tearing the very thin cuff of the tube, the Tylke forceps use a semi-closed ring at the end of the forceps which encircles the endotracheal tube but does not actually "grasp" it. This design allows the tube to be encircled and guided superiorly and anteriorly into the glottic opening and between the vocal cords while reducing the risk of inadvertently tearing the cuff. Since the Tylke forceps surround the tube, it permits either a user to push the intubation tube into the glottic opening with the forceps, or an operator could position the tube with the forceps while an assistant progresses the tube from outside the nare. Another advantage of this design is that it eliminates the need to regrasp the endotracheal tube during the intubation process. According to the manufacturer, Tylke forceps make NI up to 75% faster than intubation with other forceps while reducing cuff tearing and minimizing vocal cord trauma. The features of the Magill and Tylke forceps are illustrated in Figure 4.

While Tylke forceps have several advantages over Magill forceps for NI, the design of Magill forceps allows for several additional applications. For example, Magill forceps have become routinely used for the removal of foreign bodies from airways, even for objects as small as a safety pin [25-32]. These forceps have also been used in the placement of nasogastric tubes into the esophagus under direct visualization [33,34]. While Tylke forceps could theoretically be used for the retrieval of foreign objects, the semi-closed loop design of the distal aspect of the tongs could possibly complicate this process. Furthermore, the semi-closed loop provides more of a risk of injuring tissue since it is more sharply edged compared to the rounded clasps of the Magill forceps. From an economical viewpoint, the Tylke forceps are more expensive than the Magill forceps, and the price gap must be closed to make Tylke a more viable option than the significantly cheaper Magill forceps. While Tylke forceps may be purchased at a price of $75.00 for the disposable forceps and $109.00 for the reusable forceps, Magill forceps are available for as little as $7.00. Both forceps are commercially available in several standard sizes. (Table 1).

**Table 1:** Commercially available sizes of Magill and Tylke forceps

	Magill forceps	Tylke forceps
Pediatric/Infant	6-inch	6.25-inch
Child	7.5-inch	8-inch
Adult	9.5-inch	10-inch

The present investigation explored the time required for successful intubation using Tylke forceps compared to Magill forceps as well as in comparing the muscle activity required during the intubation process. We used surface EMG from electrodes attached to various muscle groups in the upper extremities of volunteers to record muscle activity in various muscle groups during the intubation process. Related to design and ergonomic differences in the Tylke forceps, we hypothesized that there might be measurably different efforts exerted during the placement of the endotracheal tube when using Tylke forceps compared to the Magill forceps. To our knowledge, this is the first study comparing muscle activity required during the intubation process between the two forceps.

In this initial trial, we recorded 20 total intubations. About 10 of these were performed with the Tylke forceps and 10 with the more familiar Magill forceps. An anesthesia practice mannequin was used for the NI. The intubation time was significantly faster when using the Tylke forceps compared to when using the Magill forceps. Using Tylke forceps, the EMG recordings from only the deltoid muscles demonstrated a higher overall muscle activation than Magill forceps. Whereas the EMG signal from the deltoid sensor displayed significantly less activation using the Magill than the Tylke forceps. The deltoid muscle demonstrated approximately half the muscle activation during intubation when compared to Tylke. Another important aspect of this study was that all the participants anecdotally declared little if any strain at the level of the wrist utilizing the Tylke instrument. This statement was made in comparison to greater and more significant strain on the participants' wrists when using Magill forceps. The design of Tylke suggests short-term and/or long-term benefits with its use. Given that the strain was significantly less and the time to insert the endotracheal tube was faster, suggesting a potential benefit for patients and practitioners alike.

Moreover, the efficient use of Tylke forceps with untrained participants suggests that Tylke is a more easily used tool for individuals with low- or no experience with intubation. Faced with learning both forceps, participants utilized the Tylke with significant results at a faster and more efficient rate. These participants did not have to reposition as often to recollect the endotracheal tube and could also intubate the mannequin faster. With these factors as well as an obviously lower amount of wrist strain, trained anesthetists should also be able to learn to use this tool faster and operate it even more efficiently. Furthermore, the ability to spend less time intubating paired with less wrist strain could positively impact the long-term health of someone intubating on a consistent schedule.

The limitations of this study primarily involve details about the participants. First, this study only analyzed untrained participants to analyze the two tools without any knowledge bias; however, this aspect did not analyze the long-term use of both tools compared to one another. Furthermore, the study was performed using an intubation mannequin rather than tested in a patient setting. The recording of muscle activation was also done through sensors that were attached by investigators, allowing for potential error in the placement of the scanners. Other areas of this study are also limited due to the novelty of this research and the limited amount of data, testing the Tylke forceps. Moreover, this study served as the initial research point for analyzing Tylke forceps and their validity within a clinical setting. A larger sample size, a wider range of intubation experience, and different study designs would be beneficial to further investigate the advantages and disadvantages of the two forceps.

## Conclusions

Overall, the present investigation demonstrated that greater muscle activity is used in the upper back muscles and forearm when intubating with Magill forceps, while more muscle activity comes from the muscles of the deltoid when using Tylke forceps. Magill may offer less deltoid muscle activation, while Tylke forceps allowed for fewer repositions which provided consistently faster times to intubation over Magill. When using Tylke forceps, the required muscle activation is greater than Magill forceps. However, when viewing the EMG, this activation appears to come in bursts which gives the higher RMS as opposed to the Magill EMG, which shows a lower activation required but spread out amongst the entire time of recording during intubation. While the muscle activation was greater in one of the three measured muscles while using the Tylke forceps, the intubation time was found with significant data to be faster. We feel that utilization of Tylke forceps offers improvements in the anesthetist’s fine motor control since the primary muscle activity arises from the muscles of the forearm versus the muscles of the upper arm when using Magill forceps. While this initial study had limited participants, the concept of using Tylke forceps appears to be the superior, more modern approach to NIs and represents a step forward from the traditional Magill forceps.
